# A case of Raynaud's phenomenon of toes induced by whole‐body warm stimulation

**DOI:** 10.1002/jgf2.503

**Published:** 2021-10-21

**Authors:** Takashi Akimoto, Tadashi Kobayashi, Hiroki Maita, Hiroshi Osawa, Hiroyuki Kato

**Affiliations:** ^1^ Department of General Medicine Hirosaki University School of Medicine and Hospital Hirosaki Japan; ^2^ Development of Community Healthcare Hirosaki University Graduate School of Medicine Hirosaki Japan; ^3^ General Medicine Hirosaki University Graduate School of Medicine Hirosaki Japan

**Keywords:** diagnosis, heat stress, self‐taken photograph, thermal Raynaud's phenomenon, thermal stimulation, thermal stimuli

## Abstract

Raynaud's phenomenon, induced by cold stimulation and emotional stress, is also induced by whole‐body warm stimulation. A 74‐year‐old man was referred to our department because of nocturnal toe pain from 2 years prior and immediate color change of the toes from 1 year prior when submerging himself into a warm bath. Physical examination and blood tests revealed no abnormal findings suggestive of secondary Raynaud's phenomenon. Two years later, the signs and symptoms persisted. When physicians confirm Reynaud's phenomenon, they should check for the possibility of secondary Reynaud's phenomenon. Additional research on Reynaud's phenomenon induced by warm stimulation is needed.

## Graphical abstract

Raynaud's phenomenon, induced by cold stimulation and emotional stress, is also induced by whole‐body warm stimulation. A 74‐year‐old man was referred to our department because of nocturnal toe pain from 2 years prior and immediate color change of the toes from 1 year prior when submerging himself into a warm bath. Additional research on Reynaud's phenomenon induced by warm stimulation is needed.

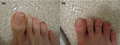

## 1 INTRODUCTION

Raynaud's phenomenon (RP) is classified as primary or secondary: primary RP is idiopathic, whereas secondary RP is caused by various underlying diseases or conditions including scleroderma and carpal tunnel syndrome.^1^, ^2^ Both primary RP and secondary RP are generally triggered by cold stimulation or emotional stress^2^ and improve with warm stimulation. Some reports indicate that secondary RP is induced by warm stimulation;^3^, ^4^ however, there have been no reports of primary RP induced by warm stimulation. Here, we report a case of suspected primary RP of the toes induced by whole‐body warm stimulation.

## 2 CASE

A 74‐year‐old man with asymptomatic adrenocortical hyperplasia was referred to our outpatient department. He reported that over the last year, his right second through fourth toes turned white immediately after entering the bath and returned to normal within a minute thereafter. His toe did not change color in the bathroom or at night. The frequency of a color change in his toes remained the same in different seasons. In addition, he reported dull pain in his toes when he woke up to go to the bathroom a few hours after going to bed which started 2 years ago. His feet were covered and always warm, while he slept. He had no history of toe trauma or toe paralysis and no history of taking oral medications, which could cause Raynaud's phenomenon.^5^ He reported that he had quit smoking in his 30s and never worked in an occupation that induced vibrational disease.

His vital signs were as follows: blood pressure, 157/99 mmHg; pulse rate, 77 bpm; and axillary body temperature, 35.9°C. On physical examination, his toes had normal color, capillary refill was rapid bilaterally within 2 s, and the bilateral dorsal pedis arteries were palpable. His blood test results were within normal limits for the following: white blood cell count (including differential leukocyte count), hemoglobin concentration, blood platelet count, erythrocyte sedimentation rate, C‐reactive protein concentration, total serum protein concentration, serum albumin concentration, hemoglobin A1c, low‐density lipoprotein cholesterol, high‐density lipoprotein cholesterol, triglyceride, thyroid function tests, proteinase 3 anti‐neutrophil cytoplasmic antibodies (ANCA), myeloperoxidase ANCA, anti‐cardiolipin antibody, rheumatoid factor, anti‐nuclear antibody, anti‐RNA polymerase III antibody, and anti‐U1‐ribonucleoprotein antibody. He had no hepatitis C antibodies. His right and left ankle‐brachial indices were 1.17 and 1.16, respectively. He had no abnormal findings on thoracoabdominal‐pelvic plain and contrast‐enhanced computed tomography or upper and lower gastrointestinal endoscopy.

**FIGURE 1:**
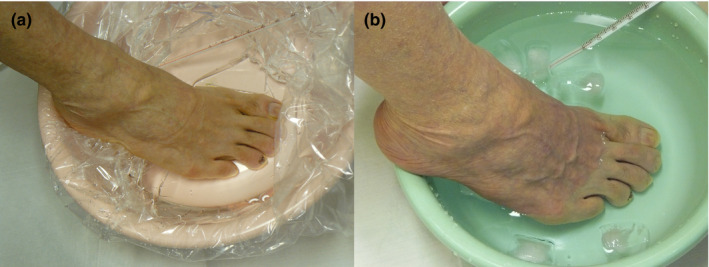
(A) Patient's right foot in warm water (42°C). (B) Patient's right foot in cold water (6°C)

**FIGURE 2:**
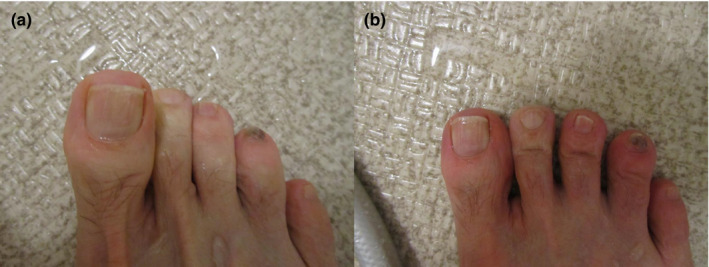
(A) Color tone of the patient's right foot immediately after immersion. (B) Color tone of the patient's right foot after continued bathing

We suspected that RP was responsible for the whitening of the toes. We confirmed this in an outpatient examination room by first immersing the right toes in warm water at 42°C, the same temperature as the water during normal bathing, and subsequently in cold water at 6°C (Figure 1). However, the color tone of his toes did not change. At a later date, we asked him to take a picture of the change in color of the right toes immediately after putting his body in the bathtub. We confirmed that the right second through fourth toes turned white, and that the color tone improved upon continual bathing for about one min (Figure 2).

No findings or data suggested secondary RP. However, the diagnostic criteria for primary RP as outlined in the International Consensus Criteria for the Diagnosis of RP published in 2014^6^ were not met because the reported symptoms were not caused by cold stimulation. Nevertheless, three other criteria (negative findings suggestive of secondary causes, no history of existing connective tissue disease, and negative or low anti‐nuclear antibody titer) were met. Therefore, the patient was suspected of primary RP. His symptoms induced no restrictions to his daily life. Two years after his first visit to our department, his toe pain had decreased in frequency and intensity. No symptoms or findings appeared to be indicative of secondary RP.

## 3 DISCUSSION

Our case report revealed two important findings: (1) RP can be induced by whole‐body warm stimulation, and (2) when RP is identified, we should confirm that there are no other diseases that can be confused with RP.

Raynaud's phenomenon is generally induced by local or general cold stimulation or strong emotional stress, or other factors.^7^ Some reports indicate that secondary RP can be induced by warm stimulation.^3^ However, the only existing reports of secondary RP induced by warm stimulation are from Japan.^3^, ^4^ One reason for this may be that in Japan, the custom is to bathe by submerging oneself in warm water, thus providing more opportunities to experience this phenomenon compared with other countries that may shower, for example. It is important to exclude secondary RP because if secondary RP is observed, treatment of the underlying disease is necessary. In our case, although we were not able to confirm capillaries in the nailfold area, the results of blood tests and physical examination indicated that the possibility of secondary RP was low. We therefore determined that the patient might have had primary RP. The mechanism of the appearance of localized RP due to whole‐body warm stimulation is currently unknown. Vascular endothelial cell dysfunction has been associated with hypertension and aging,^8^ both present in our patient; however, there was no evidence that the patient had vascular endothelial cell dysfunction. Overall, the pathological mechanism requires further study.

Several diseases have been reported to be confused with RP because they can be present with RP at the same time.^9^ Erythromelalgia is characterized by a skin rash at the ends of the extremities that is exacerbated by warm stimulation; however, RP is considered to be on the same spectrum as this disease because of the abnormal vascular response.^10^ In our case, the results of the blood tests did not suggest myeloproliferative disease.

If RP is confirmed by warm stimulation, the physician should investigate the possibility of secondary RP. Additional research on Reynaud's phenomenon induced by warm stimulation is needed.

## CONFLICT OF INTERESTS

The authors have stated explicitly that there are no conflicts of interest in connection with this article.

## INFORMED CONSENT

Written informed consent was obtained from the patient for publication of this case report.
